# Expression of oestrogen receptor beta (ERβ1) protein in human breast cancer biopsies

**DOI:** 10.1038/sj.bjc.6600035

**Published:** 2002-01-21

**Authors:** P T K Saunders, M R Millar, K Williams, S Macpherson, C Bayne, C O'Sullivan, T J Anderson, N P Groome, W R Miller

**Affiliations:** MRC Human Reproductive Sciences Unit, 37 Chalmers Street, Edinburgh, EH3 9ET, UK; Breast Research Unit, Paderewski Building, Western General Hospital, Crewe Road, Edinburgh EH4 2XU, UK; Department of Pathology, University of Edinburgh, Western General Hospital, Edinburgh, UK; School of Biological Sciences, Oxford Brookes University, Oxford OX3 0BP, UK

**Keywords:** oestrogen receptor, ERβ, monoclonal, breast

## Abstract

Oestrogen action is mediated via specific receptors that act as ligand-activated transcription factors. A monoclonal antibody specific to the C-terminus of human oestrogen receptor beta has been characterized and the prevalence of expression of oestrogen receptor beta protein investigated in a well defined set of breast cancers. Reverse transcription-polymerase chain reaction analysis of RNA from tissue biopsies detected oestrogen receptor beta in all samples examined. The anti-oestrogen receptor beta antibody cross reacted specifically with both long (∼59 Kd) and short (∼53 Kd) forms of recombinant oestrogen receptor beta. Western blot analysis of breast tumours contained both forms of oestrogen receptor beta protein although in some samples lower molecular weight species (32–45 Kd) were identified. Fifty-one breast cancer biopsies were examined using immunohistochemistry; 41 (80%) were immunopositive for oestrogen receptor alpha, 48 (94%) were immunopositive for oestrogen receptor beta and 38 (74.5%) co-expressed both receptors. Expression of oestrogen receptor beta was exclusively nuclear and occurred in multiple cell types. There was no quantitative relationship between staining for the two ERs although in tumours in which both receptors were present immunoexpression of oestrogen receptor alpha was invariably more intense. The significance of oestrogen receptor beta protein expression in breast cancers to therapy remains to be determined but the availability of a well characterized antibody capable of detecting oestrogen receptor beta in archive material will facilitate the process.

*British Journal of Cancer* (2002) **86**, 250–256. DOI: 10.1038/sj/bjc/6600035 www.bjcancer.com

© 2002 The Cancer Research Campaign

## 

Until recently it was accepted that the major effects of oestrogen on the growth and development of the breast and its tumours was mediated through a single oestrogen receptor (ERα, Green *et al*, 1986). Ligand binding assays and immunohistochemical studies indicated that most breast tumours possessed such receptors and their presence was associated with the likelihood of response to endocrine therapy (McGuire *et al*, 1982; Jordan *et al*, 1988; Miller, 1996). However in 1996 an additional ER isotype, usually known as ERβ, was identified in rat (Kuiper *et al*, 1996) and human (Mosselman *et al*, 1996). Both receptors share significant sequence homology within their DNA and ligand binding domains but are encoded on different chromosomes (Enmark *et al*, 1997). Studies *in vitro* have demonstrated that although both ERα and ERβ bind oestradiol with equal affinity (Kuiper *et al*, 1997) these receptors may have differential responses to some oestrogen agonists and antagonists (Watanabe *et al*, 1997; Barkhem *et al*, 1998; Jones *et al*, 1999; Sun *et al*, 1999). Notably ERβ appears to have a higher affinity for phytoestrogens, including genestein, than does ERα (Kuiper *et al*, 1997). When present within in the same cell, ERα and ERβ have the capacity to form either homo- or heterodimers (Pace *et al*, 1997) and the proportions of the different isotypes may be critical to modulation of gene expression (Hall and McDonnell, 1999). Studies in mammary tissues of the rat have suggested that one role of ERβ may be to antagonize ERα-mediated actions in epithelial cells (Saji *et al*, 2000), a function supported by data from *in vitro* cell transfections (Hall and McDonnell, 1999).

To date studies demonstrating the expression of ERβ in breast cancer tissues have largely been confined to the demonstration of expression of ERβ mRNA (Dotzlaw *et al*, 1997; Leygue *et al*, 1998; Speirs *et al*, 1999; Vladusic *et al*, 2000). Messenger RNAs encoding variant forms of both ERα (Bollig and Miksicek, 2000) and ERβ (Lu *et al*, 1998) have been identified in breast cancers and in breast cancer cell lines and there has been considerable debate over the role of such variants in cancer progression (Balleine *et al*, 1999; Huang *et al*, 1999).

The present investigation was designed to characterize the expression of ERβ and ERα proteins in a series of 51 breast cancers; some samples were also subjected to analysis for mRNAs by RT–PCR. We have made use of specific monoclonal antibodies and used both immunohistochemistry on well-fixed tissues in which the cellular architecture has been preserved as well as Western analysis of tissue extracts. These investigations have demonstrated wide spread expression of ERβ protein and provide new information important for further exploration of the relationship between the co-expression of ERβ and ERα and the in response of breast cancers to endocrine therapies.

## MATERIALS AND METHODS

### Patients and tissue samples

Samples of breast were obtained from 51 consecutive patients presenting to the Edinburgh Breast Unit with diagnosis of breast cancer who had given informed consent for tissue to be used for research purposes. Samples were snap frozen to provide material for extraction of RNA or protein, or fixed in 10% neutral buffered formaldehyde for 16 to 24 h then stored in 70% (w v^−1^) ethanol prior to processing into paraffin wax at the Department of Pathology using standard procedures.

### Detection of ERα and ERβ by reverse transcription-polymerase chain reaction (RT–PCR)

RNA was extracted using the Tri-reagent system according to the manufacturer's instructions (Sigma, Poole, Dorset, UK), dissolved in RNase-free water and stored at −70°C. One microgram of RNA was reverse transcribed for 1 h at 42°C in a 20-μl reaction using the Superscript system (Gibco-BRL, Paisley, Scotland, UK). Upon completion of the incubation, the sample cDNAs were each diluted to a final volume of 60 μl, and 20 μl used in individual PCR reactions containing primers specific for ERα, ERβ or alpha-actin (positive control). The primers employed were as follows: human ERα (Green *et al*, 1986), forward 5′-GGCCAGTACCAATGACAAGGGAAG-3′ (nucleotides 787–811); ERα, reverse 5′-CCAGCAAGCATGTCGAAGATCTCC-3′ (nucleotides 1558–1580); human ERβ (Ogawa *et al*, 1998a), forward 5′-GTTGCGCCAGCCCTGTTAC-3′ (nucleotides 493–512); ERβ, reverse 5′-CTCGTCGGCACTTCTCTGTCTC-3′ (nucleotides 788–809); alpha-actin forward, 5′-GGAGCAATGATCTTGATCTT-3′; alpha-actin reverse, 5′-CCTTCCTGGGCATGGAGTCCT-3′. The primers used to amplify the oestrogen receptor cDNAs were chosen to span regions separated by two intronic regions. PCR reactions were carried out using ‘Hot start’ Taq polymerase (Qiagen, Crawley, West Sussex, UK) and the following cycling conditions; 96°C for 30 s, 56°C for 1 min, 72°C for 1 min, repeated for 30 cycles for ERα, similar conditions were used for ERβ except that the annealing temperature was 52°C. The expected sizes of the amplified bands were; ERα, 793 bp; ERβ, 316 bp; alpha actin 120 bp. Nine samples were analyzed.

### Antibodies

The anti-hERα mouse monoclonal antibody (code 1D5) was obtained from DAKO (Cambridge, UK). A peptide located at the C-terminus of hERβ (Mosselman *et al*, 1996) (CSPAEDSKSKEGSQNPQSQ) was used to prepare a monoclonal antibody in mice according to standard methods and positive clones were identified by ELISA using recombinant human ERβ (P2466, PanVera, Madison, WI, USA) (Saunders *et al*, 2000). This antibody has been used previously to demonstrate expression of ERβ using human ovarian tissue sections (Saunders *et al*, 2000).

### Western analysis

Two forms of recombinant human ERβ1 were obtained from Pan Vera (Madison, WI, USA). These were hERβ1 ‘short’, a ∼53 Kd form of the receptor (βs) synthesized from a cDNA (Mosselman *et al*, 1996) lacking the first potential start site for translation (Ogawa *et al*, 1998a), and hERβ1 ‘long’ (βL) the larger protein (∼59 Kd) synthesized from the full length cDNA (Ogawa *et al*, 1998a). Recombinant hERα (∼66 Kd) was also obtained from Pan Vera. Gel analysis and blotting were carried out as described previously (Saunders *et al*, 2000). Briefly, proteins were extracted from frozen biopsy specimens by rapid homogenization of tissue in denaturing/loading buffer (50 mM Tris-HCl pH 6.8, 100 mM DTT, 2% SDS, 0.1% bromophenol blue, 10% glycerol, all from Sigma). Recombinant proteins (0.5 μg lane^−1^), tissue extracts (30–50 μg total protein) and prestained protein molecular weight markers (BioRad) were separated on denaturing minigels containing an acrylamide gradient from 4 to 20% (w v^−1^) polyacrylamide (Novex, San Diego, CA, USA). Membranes were incubated overnight with the mouse monoclonal anti hERβ1 (code M9) at 1 in 500 or mouse monoclonal anti-hERα (code1D5) at 1 in 100; both the antibodies were diluted in TBST containing 5% normal donkey serum. Bound antibodies were detected using rabbit anti-mouse IgG and the ECL visualization system (Amersham, Bucks, UK) according to the manufacturer's instructions.

### Immunohistochemistry

Sections (4 μm) were mounted on Superfrost coated slides (BDH, Poole, Dorset, UK) dewaxed and rehydrated in gradient alcohols and distilled water. Endogenous peroxidases were blocked with 3% hydrogen peroxide for 10 min and sections were subjected to heat-induced antigen retrieval in 0.01 m citrate buffer, pH 6.0 (Norton *et al*, 1994) before staining with specific antibodies as outlined below.

#### Anti-ERα

All staining for ERα was carried out in the Pathology Department of the Western General Hospital. An endogenous biotin block was carried out by applying 100 μl egg white blocking solution for 30 min. Anti-ERα, (Dako) was diluted 1 in 50 in biotin diluent for primary antibodies (PBS, goat serum and d-biotin), and incubated in the sections for 60 min at room temperature. The secondary antibody, biotinylated anti-mouse Ig(Vector Laboratories) was diluted 1 : 2000, in ‘background reducing diluent’ (Dako) and applied to sections for 30 min at room temperature. The tertiary system (ABC-HRP, Dako) was applied as per manufacturer's instructions for 30 min at room temperature. The tissue was visualized by immersing sections in 3,3′-diaminobenzidine tetra-hydrochloride (DAB) for 5 min. Sections were counterstained using Mayers haematoxylin (Sigma-Aldrich, Poole, Dorset), dehydrated through gradient alcohols and mounted.

#### Anti-ERβ

Immunolocalization was undertaken as described in detail in Saunders *et al* (2000), Sections were blocked for 30 min in normal rabbit serum (NRS, Diagnostics Scotland, Carluke) diluted 1 : 4 in TBS containing 5% BSA (NRS/TBS/BSA), rinsed briefly in TBS and an avidin biotin block performed using reagents from Vector (Peterborough, UK). Anti-ERβ antibody was diluted 1 : 40 in NRS/TBS and incubated on sections overnight at 4°C. Sections were washed twice for 5 min each time in TBS and incubated with rabbit anti mouse, (Dako, Cambridge, UK) diluted 1 : 500 in NRS/TBS/BSA. Thereafter, bound antibodies were visualized by incubation with 3,3′-diaminobenzidine tetra-hydrochloride (liquid DAB cat K3468, DAKO). Sections were counterstained with haematoxylin.

Images were captured using an Olympus Provis microscope (Olympus Optical Co, London, UK) equipped with a Kodak DCS330 camera (Eastman Kodak Co., Rochester, NY, USA), stored on a Macintosh PowerPC computer and assembled using Photoshop 5.5 (Adobe, Mountain View, CA, USA).

### Quantitation of immunohistochemical staining

Quantitation was based on a scoring system reported in detail previously (Allred *et al*, 1998; Leake *et al*, 2000). This method is based on a composite additive score of intensity 0–3 and proportion of malignant epithelial cells staining 0–5. This gives a range from 0–8 for each tissue. Samples were analyzed using the SPSS package (version 10 for Macintosh; SPSS Inc, Chicago, IL, USA) and plotted as a box and whisker plot. No correlation between ERα and ERβ scores was detected.

## RESULTS

### Detection of mRNAs for ER*α* and ER*β* in breast cancer samples

**Figure 1:**
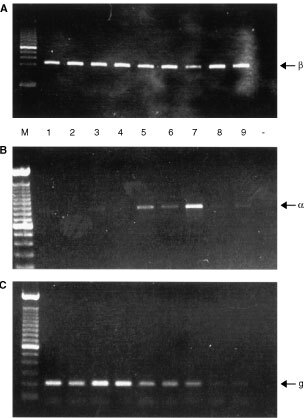
Detection of oestrogen receptor mRNAs by RT–PCR. (**A**) ERβ, (**B**) ERα, (**C**) Alpha-actin. In all panels, lane M 100 bp ladders, lanes 1–9 breast tumour samples, the negative control lane (−) contained a sample prepared without reverse transcriptase. Note that although a cDNA specific for ERβ was amplified from all samples, the amount of ERα cDNA amplified from the same sample set was highly variable.

All samples tested (*n*=9) were positive for ERβ following RT–PCR (Figure 1

). This signal always appeared greater than those for ERα and was present in both ERα positive and negative samples. Actin was amplified from all samples although the efficiency of the reaction was variable.

### Specificity of antisera and extraction of ER proteins from breast cancer biopsies

**Figure 2:**
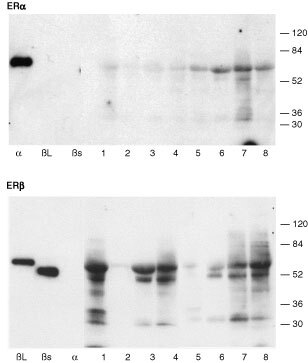
Western analysis of proteins extracted from breast cancer samples. Proteins were separated, blotted and incubated with antibodies directed against ERα (upper panel) or ERβ (lower panel). The anti-ERα antibody bound to recombinant hERα but not to recombinant hERβ (βs, βL). The anti-ERβ1 antibody bound to both long (βL) and short (βs) forms of recombinant hERβ but not to recombinant hERα (α). Proteins migrating with the same apparent molecular size as recombinant ERα (α, upper panel, arrowhead) were detected in all breast samples (lanes 1 to 8, note identical samples were used for both gels and are loaded in the same order). In sample numbers 6 and 7 additional lower molecular weight forms of ERα were present. Variable amounts of ERβ proteins were detected in the same samples. Proteins migrating with the same apparent molecular size as both long and short forms of ERβ proteins (arrowheads) were detected in breast sample numbers 1, 3, 4, 6, 7, 8; additional lower molecular weight variants were present in these same extracts but samples 2 and 5 lacked significant levels of ERβ.

On Western blots (Figure 2

) antibodies directed against ERα and ERβ bound to either recombinant ERα or recombinant ERβ protein depending upon the isotype to which they were directed. These results were consistent with previously published data (Saunders *et al*, 2000); no binding of the ERβ specific monoclonal to ERα was observed (Figure 2, lower panel, lane α). The anti-hERβ monoclonal that was directed against a peptide at the C-terminus of hERβ bound to both short (Mosselman *et al*, 1996) and long (Ogawa *et al*, 1998a) forms of ERβ. This result is consistent with data that has demonstrated that the difference in size of the long and short forms of ERβ is due to use of alternative start sites for translation within the full length mRNA and that the C-termini of both proteins are identical.

Tissue biopsied from eight tumours, that were histologically shown to be cancers, were also examined. The predominant form of the ERα protein (Figure 2, upper panel) extracted from all biopsies migrated with an apparent molecular size (∼66 Kd) identical to recombinant ERα run in a parallel lane (α). In only two samples (lanes 6 and 7) did we see evidence of expression of shorter/variant ERα proteins.

The amount of ERβ protein detected in extracts from cancer biopsies was highly variable (Figure 2 lower panel). It was notable that in six of the eight samples proteins migrating with apparent molecular sizes corresponding to both long (∼59 Kd) and short (53 Kd) ERβ were present. We have found that this antibody recognizes ERβ protein extracted from human ovary, prostate (Saunders *et al*, 2000) endometrium and testis and human cell lines (MCF-7, Ishikawa, unpublished observations). In breast tumour samples that appeared to contain high levels of expression of full length ERβ (numbers 1, 3, 4, 7, 8) several lower molecular weight protein species with apparent molecular weights from 32 to 45 Kd were detected.

### Immunolocalization of oestrogen receptors

**Figure 3:**
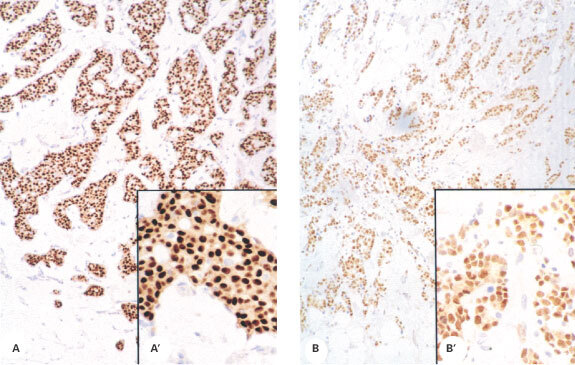
Immunoexpression of ERα in human breast cancers. Nuclear expression of ERα was largely confined to malignant epithelium in the 40 samples in which it was detected; intensity was variable. (**A**) example of intense immunostaining (sample code 5580); (**B**) sample code 5667, magnification ×20, insets A′ and B′ show higher power magnification of the same tissue samples.

Typical examples of immunostaining for ERα and ERβ are shown in Figures 3

and 4

**Figure 4:**
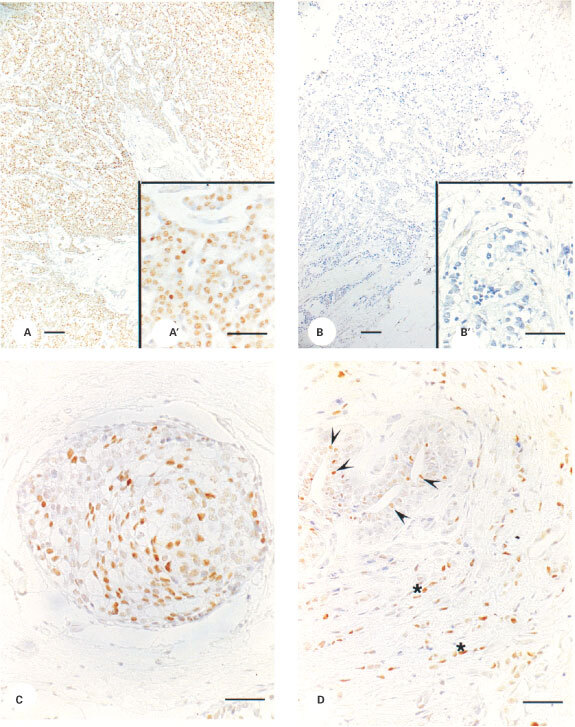
Immunoexpression of ERβ in human breast tissues. Nuclear expression of ERβ protein was detected in 94% of the samples examined. (**A**,**B**) show examples of immunopositive (**A**, code 5580) and immunonegative (**B**, code 5667) staining of malignant tissue. Expression of ERβ was also noted in non-invasive ductal cancer (**C**) and in epithelial (**D**, arrowheads) and stromal (**D**, asterisks) cells in areas of breast not associated with malignant growth. (**A**,**B)**, Magnification ×10, bar=100 μM, insets A′ and B′ are from the same tissues as **A** and **B**, magnification ×40, bar=50 μM. (**C**, **D**) Magnification ×40, bar=50 μM.

respectively. Staining for ERα (Figure 3) was predominantly nuclear and almost exclusively restricted to malignant epithelium (insets A′ and B′) in this tissue series. Note that the malignant tissues illustrated in Figure 4A,B are the same as those in Figure 3A,B (codes 5580 and 5667 respectively) and clearly illustrate that ERα expression (Figure 3) can occur in the presence (Figure 4A) or absence (Figure 4B) of ERβ. Expression of ERβ was almost exclusively nuclear and often appeared granular and heterogeneous (Figure 4A′). Expression of ERβ was noted in a wider range of cells than was ERα and was found in non-malignant components of the tumour including normal glandular elements (Figure 4D arrows), blood vessels, adipose tissue and stromal cells (asterisks) as well as in non-invasive intraduct cancers (Figure 4C).

### Quantitation of immunohistochemical staining

**Figure 5:**
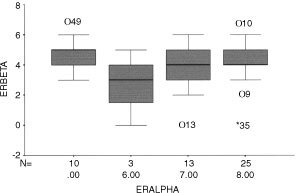
Quantification of immunoexpression for ER isotypes. Box and whisker plot summarising the relationship between score for ERα (x-axis) and ERβ (y-axis) for each sample. Solid horizontal line shows the median for the data, the top of the box the 25th percentile, the bottom the 75th percentile and the additional lines the range of the data. Note that there were no samples with an ERα score of 1 to 5.

Most of the tumours (48 out of 51) displayed staining for ERβ in malignant epithelium with a range of scoring between 2 and 7 (median score 4.5). ERα staining was found in 41 out of 50 tumours with a range of scoring between 6 and 8 (median score 7.5). Quantitatively it was possible to identify ERα-positive, ERβ-positive tumours (38 out of 51, Figures 3A and 4A) as well as ERα-positive, ERβ-negative tumours (3 out of 51, Figure 3B compared with Figure 4B; 2 out of 51). ERα-negative, ERβ-positive tumours were detected (10 out of 51) but we observed no double negatives. There was no quantitative relationship between immunohistochemical scores for ERα and ERβ (Figure 5

).

## DISCUSSION

Many breast cancers, like the normal tissue from which they are derived, appear sensitive to oestrogens. The major action of oestrogen appears to be mediated by specific receptor proteins that act as nuclear transcription factors. Until recently, studies have concentrated on the ERα member of the family and these have clearly demonstrated the involvement of the protein in maintaining the growth of hormone sensitive tumours. As a consequence ERα measurements have been used to select patients for endocrine therapy and the protein has become a therapeutic target by which to treat patients with breast cancer. Nevertheless there have been paradoxical observations such as tumours regressing following endocrine deprivation therapy in apparently ERα negative disease. Oestrogen responses in ERα knockout mice and the differential effects of anti-oestrogens in tissues and tumours were also unexplained.

Our ability to correlate ER status with outcome of therapy has been complicated by the finding of a second oestrogen receptor (ERβ) which can bind oestrogens including oestradiol and tamoxifen with high affinity (Kuiper *et al*, 1996, 1997; Mosselman *et al*, 1996). As a result there has been a major effort to delineate the role of ERβ in the natural history of breast cancer. Many papers have reported that the mRNAs for both ERα and ERβ are expressed in breast cancer cell lines (Watanabe *et al*, 1997; Moore *et al*, 1998; Vladusic *et al*, 2000), in breast cancer tissue (Dotzlaw *et al*, 1997) and in the normal human and rodent mammary gland (Moore *et al*, 1998; Saji *et al*, 2000). Studies that have compared levels of expression of the mRNAs encoding the two receptors have reported that the amount of ERβ mRNA does not appear to be correlated with that of ERα (Dotzlaw *et al*, 1997; Iwao *et al*, 2000; Vladusic *et al*, 2000) consistent with expression of the receptors by different genes (Enmark *et al*, 1997). Some studies have reported that up-regulation/over expression of ERβ mRNA may be correlated with development of oestrogen-independent tumour growth and a poor prognosis (Speirs *et al*, 1999; Iwao *et al*, 2000).

Modelling studies using ERα have defined the amino acids within the protein which interact with natural as well as synthetic oestrogens and anti-oestrogens (Ekena *et al*, 1997). The major determinants of ligand binding are conserved between ERα and ERβ consistent with their ability of both to bind oestradiol (Kuiper *et al*, 1997). Barkhem *et al* (1998) have used cell lines stabily transfected with either ERα or ERβ to test the affinity and potency of widely used anti-oestrogens including tamoxifen, raloxifine and ICI 164,384 and concluded that the ligand binding cavity of ERβ is more different to that of ERα than can be anticipated from the primary sequence. Recently novel non-steroidal ligands that show subtype specific binding affinity and transcriptional potency have been identified (Sun *et al*, 1999) and ligand-dependent differences in the ability of ERα and ERβ to recruit co-activators following exposure to xenoestrogens described (Routledge *et al*, 2000). ER-driven gene activation can be determined by the formation of homo- or hetero-dimers, the cell type, and whether the ligand-activated receptors bind to a promotor containing ERE or an AP-1 site (Watanabe *et al*, 1997; Jones *et al*, 1999). Furthermore the experience with studies on ERα has been that mRNA is not necessarily translated into protein make it essential that assays for ERβ are performed at the level of protein.

The monoclonal antibody used to detect ERβ in the present study was raised against a peptide at the C-terminus of human ERβ1 (Mosselman *et al*, 1996; Moore *et al*, 1998). This peptide is not conserved in any of the ERβ variants formed by alternative splicing of the F domain of the protein (Moore *et al*, 1998; Ogawa *et al*, 1998b) and does not recognize recombinant ERβ2/βcx on Western blots (unpublished observations). Similarly Western blotting indicated that the monoclonal antibody identified ERβ but not ERα in breast cancers. Most of the ERβ1 protein detected in the extracts from the breast cancers migrated with the same apparent size as the ‘long’ and ‘short’ forms of recombinant ERβ1, which are formed by translation from different ATGs in the mRNA (Mosselman *et al*, 1996; Ogawa *et al*, 1998a). We did not detect proteins corresponding in size to those that could be translated from mRNAs deleted in exons 5 or 6 (Lu *et al*, 1998; Brandenberger *et al*, 1999) predicted to be 16.8 and 13 Kd respectively. The most prominent proteins other than full length ERβ1 migrated between 30 and 36 Kd these could represent use of alternative start sites, translation from an exon 2 deleted mRNA (∼35 Kd) or translation of protein from mRNA deleted for both exons 5 and 6 (AF074599) which is predicted to be ∼43 Kd (short) or ∼49 Kd (long) from the mRNA sequence. It is notable that mRNAs corresponding to alternatively spliced forms of ERβ have been detected in breast cancer tissues and cell lines (Lu *et al*, 1998; Moore *et al*, 1998; Vladusic *et al*, 1998; Iwao *et al*, 2000) as well as in normal human tissues (Ogawa *et al*, 1998b; Scobie *et al*, 2001). Furthermore, monoclonal antibodies directed against the N terminus of ERβ have detected expression of proteins other than full length ERβ in breast cancer cell lines (Fuqua *et al*, 1999) which might have been formed by translation of alternatively spliced mRNAs. During the course of the present study we found that recombinant ERβ proteins (both from commercial sources and prepared in house) degrade if subjected to a single freeze-thaw cycle or following prolonged storage even at low temperatures (−70°C). Therefore although considerable attention was paid to extraction of the breast tumour samples and to the storage of extracts we believe that the most likely explanation for the lower molecular weight bands identified in samples containing the highest levels of ERβ1 is that these are breakdown products of the full length protein which have formed during handling of the protein extracts.

We have used our ERβ1 specific monoclonal antibody to immuno-localize ERβ1 in a series of breast cancers as well as in other human and primate tissues (Saunders *et al*, 2000; Scobie *et al*, 2001). The present study has demonstrated the presence of ERβ1 in cell nuclei not only the malignant epithelium but also non-malignant elements of most breast cancers. The qualitative and quantitative expression of ERβ was independent of that of ERα. We have observed that ERβ1 was also expressed in multiple types of non-cancer cells within the breast tissue and this will therefore further complicate the assessment of ERβ status. For example, methods such as RT–PCR or Western blotting which use tissue extracts may contain a contribution from cells other than those derived from the malignant component of the tumour. It will therefore be important to quantify expression in different compartments of the breast separately. This precludes the simple use of Western and Northern blotting together with other technologies in which tissue is homogenized and extracted.

Whilst our studies were being written up three reports describing immunolocalization of ERβ to breast cancer samples were published. Mann *et al* (2001) used a rabbit polyclonal antibody directed against the N-terminus of human ERβ on formalin fixed samples; on the Western blot shown in their article multiple bands are shown, the most prominent of which appeared shorter than the recombinant standard and this may reflect degradation of protein in their extracts or non-specific reactivity of the antibody used. In their paper immunopositive staining of human breast cancer for ERβ was present in 66 and 70% of the two sets of samples reported but no mention was made of immunopositive staining of cells other than those of the malignancy. The authors mentioned the potential cross-reactivity of their antibody with isoforms of ERβ including ERβcx (Ogawa *et al*, 1998b) which will not occur with the antibody used in the current study. It is notable that the polyclonal rabbit antibody used by Omoto *et al* (2001) is raised to an identical part of the ERβ1 protein to our monoclonal and we would therefore expect similar results to our own. In their study they used frozen sections of tissue and found that only 59% (52 out of 88) were positive for ERβ, with only 38% of the ERα negative samples expressing the ERβ subtype. This proportion is much lower than in the current study or in the tissue set studied by Jarvinen *et al* (2000) who used frozen sections fixed briefly with Zamboni's, and found 60% of cancers contained ERβ1 positive cells using a commercial polyclonal antibody raised to the same region of the protein. The need to use frozen sections clearly limits the utility of these antibodies and highlights an important difference with the reagent used in the present study which appears capable of identifying ERβ1 in material fixed by formalin, methacarn (unpublished observations) or Bouins (Saunders *et al*, 2000). In studies using fixed samples from human tissues including ovary, placenta, vas deferens, testis and endometrium we have used monoclonal and polyclonal antibodies to localize ERβ proteins (Saunders *et al*, 2000; Critchley *et al*, 2001; Scobie *et al*, 2001). In all cases we find the protein to be nuclear in location in agreement with the findings using fixed tissues of human breast (present study) the only exceptions being dividing cells, and some myoid cell types where background staining of the cytoplasm associated with the secondary antibodies was a problem. We have detected cytoplasmic staining using some commercial anti ERβ antibodies especially those that have not been affinity purified and with some secondary antibodies especially those raised in goats (unpublished observations). These findings may explain some of the cytoplasmic staining seen in the figures published by others (Jarvinen *et al*, 2000; Mann *et al*, 2001; Omoto *et al*, 2001).

In conclusion, we believe that to assess the responsiveness of breast cancers to oestrogenic and anti-oestrogenic stimuli it will be necessary to measure both ERα and ERβ at the level of protein. The presence of ERβ in both malignant and non-malignant components of breast tumours means that assessments in individual compartments may also be required. This approach is being utilized in our ongoing studies.
